# House dust mite sublingual allergen immunotherapy tablet is safe and well-tolerated in Dutch clinical practice

**DOI:** 10.3389/falgy.2024.1355324

**Published:** 2024-02-29

**Authors:** Žana Tempels-Pavlica, Mark C. J. Aarts, Paco M. J. Welsing, Akke-Nynke van der Meer, Leonard P. van der Zwan, Elena Uss, André C. Knulst

**Affiliations:** ^1^ Department of Allergology, Diakonessenhuis, Utrecht, Netherlands; ^2^ Department of Otorhinolaryngology, Jeroen Bosch Hospital, ‘s-Hertogenbosch, Netherlands; ^3^ Department of Rheumatology & Clinical Immunology, University Medical Centre Utrecht, Utrecht, Netherlands; ^4^ Department of Pulmonary Diseases, Medical Centre Leeuwarden, Leeuwarden, Netherlands; ^5^ Medical Department, ALK-Abelló BV, Almere, Netherlands; ^6^ Department of Dermatology and Allergology, University Medical Centre Utrecht, Utrecht University, Utrecht, Netherlands

**Keywords:** allergic rhinitis, Control of Allergic Rhinitis and Asthma Test (CARAT), house dust mite (HDM), safety, sublingual immunotherapy tablet, non-interventional study, general medicine, outpatient clinic

## Abstract

**Background:**

Half (49%) of clinically diagnosed allergic rhinitis (AR) patients are sensitized to house dust mite (HDM). If allergen avoidance and symptomatic medication fail, allergen immunotherapy may be indicated.

**Objective:**

We investigated safety and tolerability of HDM-sublingual immunotherapy by HDM-SLIT tablets in Dutch daily clinical practice.

**Methods:**

Daily intake of 12 SQ-HDM SLIT-tablet was investigated in a prospective, multicenter, observational study (EUPAS43753). It comprised 4 consultations in 1 year. Data on safety, tolerability, treatment satisfaction, symptomatic medication, compliance, and clinical effectiveness (Control of Allergic Rhinitis and Asthma Test; CARAT) were collected. Descriptive and longitudinal regression data analysis were performed.

**Results:**

Adult patients (*n* = 415), mean (SD) age 36.6 (12.2) years, 61.4% female and 36% asthmatic were included. The preponderance (65.1%) experienced adverse events (AEs). These, mostly mild (67%), AEs comprised: oral allergic reactions (58.6%), respiratory (12.4%) and gastrointestinal symptoms (9.4%). Sixty (14.5%) patients stopped due to AEs and 76 (18.3%) for non-AE reasons. CARAT scores improved clinically significant by 6 points and symptomatic medication use decreased from 96.1% to 77.4%. Most patients (74.5%) tolerated the treatment and were compliant (>86.5%). The majority of patients (62.4%) and investigators (69.4%) were satisfied with treatment.

**Conclusions:**

HDM SLIT-tablet is a safe and well-tolerated AR treatment. AEs occur often but are mostly mild and decreasing during the first year. CARAT scores improved and symptomatic medication use decreased suggesting better control of AR with treatment. Compliance, tolerability, and treatment satisfaction are good. However, patient follow-up and compliance remain important points of attention when initiating treatment.

## 1 Introduction

Allergic rhinitis (AR) affects 17%–29% of the adult population in Europe (1), thereby constituting a serious public health problem. An incidence rate of AR of approximately 9 per 1,000 patient-years has been reported for children as well as for adults in Dutch general practices (2). In addition, allergy to house dust mites (HDM), generally induced by *Dermatophagoides (D) pteronyssinus* or *D. farinae*, is the most common inhalant allergy (3) with sensitization in 49% of subjects with a clinical diagnosis of AR in Western Europe. Indicative for the importance of house dust mite in allergy is its association with co-morbidities. In general, 25% of AR patients has asthma and about 50% of asthmatic patients has rhinitis (4–7). However, the concomitant prevalence of asthma and AR in a patient appears to be more frequent in HDM sensitized patients. In a Dutch study even 92% of HDM sensitized allergic patients with asthma had AR as well (8).

Exposure of the epithelium of different organs (e.g., eyes, nose and lungs) in HDM sensitized individuals can result in atopic complaints. Mite fecal particles are the primary source of mite allergens together with exoskeleton (9).

Allergen immunotherapy (AIT) has been around for more than a century (10). The high dose of allergen administered in AIT changes the causal immunological response in an allergic patient when exposed to a specific allergen and thus reduces allergy symptoms when subsequently exposed (10, 11). Symptomatic medication may suppress mild AR, but symptomatic medication alone may be insufficient treatment in a large number of patients with moderate or severe AR (12). Therefore, AIT may be added to symptomatic treatment for an additional and sustained effect on reducing AR symptoms, being a curative rather than symptomatic treatment (13–18).

Several clinical trials have shown that treatment with HDM SLIT-tablet immunotherapy effectively reduces symptoms associated with HDM AR with or without asthma. Hence, treatment with HDM SLIT-tablet has become common practice (14–19). However, most data concerning tolerability, side effects and compliance have been obtained in clinical trials and not in a daily clinical practice setting. While RCTs have high internal validity and are needed to demonstrate a favorable risk/benefit profile, the controlled clinical trial setting with patient selection based on in- and exclusion criteria may impact the generalizability of the results to daily practice.

To assess the general applicability of the efficacy and safety data collected in randomized controlled trials (RCT's), we set out to conduct a complementary multi-center, observational study in outpatient clinics and general practices. The objectives of the study were to assess the safety, tolerability, treatment satisfaction, compliance, and clinical effectiveness of HDM SLIT-tablet treatment, when prescribed as part of regular clinical practice.

## 2 Methods

### 2.1 Study participants

Four hundred fifteen (415) patients (age 18–65 years) with HDM-induced AR with or without asthma were recruited from 71 general practices or outpatient clinics of allergologists, dermatologists, Ear-Nose-Throat (ENT) specialists or pulmonologists in the Netherlands between September 2017 and March 2019. A patient was diagnosed with HDM allergy, when having a positive skin prick test to HDM extract or allergen specific HDM IgE level of 0.35 IU/mL or higher next to a relevant clinical history. The decision to initiate treatment with HDM SLIT-tablet was made at the discretion of the physician. Key discontinuation criteria were patient-based decision or treatment-related adverse event (AE). The study was approved by the Dutch Clinical Research Federation/nWMO Advisory Committee Twente (no. NWMO17.04.017) and the applicable ethics committees and institutional review boards. All patients gave written informed consent. The study complies with the Declaration of Helsinki (20).

### 2.2 Study design

This is a non-interventional, prospective, multi-center, observational study. Data were collected and recorded during three patient visits and one consultation by phone. The first visit included on-site administration of HDM SLIT-tablet and collection of baseline characteristics. The first visit was followed by a phone interview one week later. The second visit followed three months and the final visit 1 year after the initial visit, respectively. During each visit the allergic symptoms, Control of Allergic Rhinitis and Asthma Test (CARAT) questionnaire (21), (change in) concomitant medication, lung function measurements (only if indicated according to the treating physician) and safety evaluations (AEs/SAEs) were recorded.

### 2.3 Collection, recording and reporting of adverse events

All safety data were assessed by the treating physician. Standard definitions were used for adverse event, seriousness, and outcome (22). Relatedness was defined as either “Possible’’: a causal relationship is conceivable and at least reasonably possible; or “Unlikely’’: the event is most likely related to a different etiology than the medicinal product. AEs with unclear causality were categorized as possibly related. Severity was defined as “Mild’’: No or transient symptoms, no interference with the patient's daily activities; “Moderate’’: Marked symptoms, moderate interference with the patient's daily activities; or “Severe’’: Considerable interference with the patient's daily activities, unacceptable.

Safety data were described by the number and proportion of patients with any AE, overall and per type of AE (oral allergy reactions, GI reactions, airway reactions, lower airways reactions, upper airway reactions, skin reactions, general reactions, or other reactions) and the number of patients with any moderate or severe AE. The median number (with IQR: 25th and 75th percentile Q1;Q3) of AEs experienced per patient in those experiencing any AE was also calculated. All analyses were performed separately per study visit. Safety data solicited were all serious adverse events (SAE), all causal AEs, and AEs of special interest (22). Any unsolicited safety data reported by physicians were also included. All AEs reported were categorized by preferred term and system organ class (Medical Dictionary for Regulatory Activities versions 23.0).

Furthermore, the median number of AEs and the frequency of patients in specific subgroups [patients having multiple (major) allergies, concomitant asthma, and patients that are not compliant with treatment or patients taking concurrent immunotherapy] were described per visit.

### 2.4 Compliance assessment

Treatment compliance was recorded at visits 1, 2, 3 and 4 as estimated by treating physician and categorised by compliance with treatment (1 tablet per day) since last visit: ≥80% of days; ≥50%–79% of days; ≤50% of days. We considered a compliance of ≥80% of days as good compliance.

### 2.5 Tolerability and satisfaction

A final brief evaluation was part of the study when patients completed the study or if they discontinued early. Perceived tolerability (very well tolerated, well tolerated, moderately tolerated, poorly tolerated) and treatment satisfaction (very satisfied, satisfied, not satisfied, very dissatisfied) were reported by patients and their treating physicians.

### 2.6 Study treatment

In this study, patients were treated with HDM SLIT-tablet (ACARIZAX®; 12-SQ-HDM sublingual lyophilizate immunotherapy ALK-Abelló A/S, Hørsholm, Denmark) (14–16). HDM SLIT-tablet is approved for the treatment of HDM induced AR by the Dutch authorities since July 2016 and reimbursed as of October 2017. HDM SLIT-tablet is a lyophilisate containing standardized allergen extract from two house-dust-mite species, *D. pteronyssinus* and *D. farinae* (23). The first dose was self-administered under medical supervision, and subjects were monitored for 30 min after first intake. Subsequent doses were self-administered at home. The tablet was to be placed under the tongue and allowed to remain there until dissolved. Subjects were advised not to swallow during the first minute after administration, food and beverages were not allowed for 5 min thereafter. The study duration was one year.

## 3 Statistical analysis

Regarding safety, the frequencies, and proportions of possibly treatment-related adverse events (AEs) were calculated. All AEs having at least a possible relation with the study drug were described (details see supporting information). Of all AEs reported the frequency and proportion per severity category, course, outcome, and drug adjustment in response to the AE were calculated.

To test whether the occurrence of any AE changed over time, the occurrence of any AE at 1 week, 3 months and 1 year was compared to the occurrence shortly after the first administration using multilevel modelling to account for the repeated observations within patients.

The significances of differences in frequency of AEs in specific subgroups [patients having multiple (major) allergies, concomitant asthma, and patients that are not compliant with treatment or patients taking concurrent immunotherapy] compared to patients without the respective specific capacity were analyzed by Mann–Whitney tests.

Treatment compliance was estimated and categorized by treating physician: 100%–80% (compliant), between 50% and 80%, or less than 50% and described per visit [*n* (%)].

Treatment satisfaction experienced by patients and observed by physicians (very satisfied, satisfied, unsatisfied, very unsatisfied) and treatment tolerability (very good, good, moderate, poor) were described as proportions. Mean and median values were reported with standard deviation (SD) or interquartile range (25%–75%; IQR), respectively. A *P*-value <0.05 was considered significant in all analyses.

### 3.1 Post hoc clinical effectiveness analyses

The questions of the CARAT questionnaire were either administered to the patient during a visit or were asked by the physician during the phone call. The CARAT questionnaire is a validated useful tool for facilitating optimal control of both asthma and allergic rhinitis simultaneously (24, 25) and is included in Dutch guidelines (26). The current adult version consists of 4 questions on weekly frequency of AR symptoms, 5 questions on weekly frequency of asthma symptoms and 1 question about extra symptomatic medication use (available at caratnetwork.org) (25, 27). CARAT scores vary from 0 points (worst) to 30 points (best) outcome (27). A CARAT score of 24 points or lower is considered poor control (27). The minimal clinical important difference (MCID) for CARAT scores was established at a 4 points difference by using the Global Rating of Chance and standard error of the mean in a Dutch cohort in 2015 (24). Changes equal or more than MCID, with a *P *< 0.05 for difference in means, were considered indicative for clinical effectiveness.

## 4 Results

### 4.1 Patient population

Patient's demographics and baseline characteristics are shown in Table 1. The mean age of the predominantly female population (61.4%) was 36.6 (SD: 12.2) years. Average time between onset of AR symptoms and initiation of SLIT treatment was 8 (IQR: 1:17) years. One third of patients (36.1%) had concomitant allergic asthma. The proportion of polysensitized patients was 79%. Main co-sensitizations were grass pollen (76.5% of patients), tree pollen (54%), and animal dander (48.5%).

**Table 1:** Baseline characteristics of patients.

Characteristic	Total *N* = 415	Completed study *n* = 277	Discontinued *n* = 138	*P*-value for comparison
Age, years^a^	36.6 (12.2)	37.5 (12.2)	34.9 (12.0)	0.0388
Weight, kg^a^	77.0 (15.4)	77.1 (15.5)	77.0 (15.3)	0.9639
Height, cm^a^	173.8 (9.6)	174.1 (9.5)	173.1 (9.6)	0.2933
BMI^a^	25.5 (4.5)	25.4 (4.5)	25.8 (4.7)	0.3918
Female sex^c^	255 (61.4)	168 (60.6)	87 (63.0)	0.7151
Treated by general practitioner^c^ (vs. specialist)	116 (28.0)	68 (24.5)	48 (34.8)	0.03821
HDM-induced AR^c^	415 (100)	277 (100)	138 (100)	–
Years with HDM-induced AR^b^	8 (1;17)	8 (1;18)	7 (1;9.7)	0.1323
Asthma^c^	150 (36.1)	105 (37.9)	45 (32.6)	0.2899
Family history of allergy^c^	152 (27.2)	95 (34.4)	57 (41.3)	0.1978
Co-allergy status^c^				0.0737
Mono sensitized (only HDM)^c^	87 (21.0)	60 (21.7)	27 (19.6)	
One co-allergy^c^	100 (24.1)	57 (20.6)	43 (31.2)	
Two co-allergies^c^	124 (29.9)	91 (32.9)	33 (23.9)	
Three or more co-allergies^c^	104 (25.1)	69 (24.9)	35 (25.4)	
Type of co-allergies^c^				
Tree pollen^c^	177 (54.0)	123 (44.4)	54 (39.1)	0.3586
Grass pollen^c^	251 (76,5)	166 (59.9)	85 (61.6)	0.8254
Animal dander^c^	159 (48.5)	110 (39.7)	49 (35.5)	0.4698
Plant pollen^c^	10 (2.4)	8 (2.9)	2 (1.4)	0.5071
Fungus spores^c^	13 (3.1)	8 (2.9)	5 (3.6)	0.7667
Food^c^	34 (8.2)	24 (8.7)	10 (7.2)	0.7594
Other^c^	49 (11.8)	29 (10.5)	20 (14.5)	0.3006
Current immunotherapies use other than HDM SLIT-tablet^c^				0.05921
No other immunotherapy^c^	355 (85.5)	234 (84.5)	121 (87.7)	
One immunotherapy^c^	47 (11.3)	37 (13.4)	10 (7.2)	
Two immunotherapies^c^	13 (3.1)	6 (2.2)	7 (5.1)	

AR, allergic rhinitis; BMI, body mass index; HDM, house dust mite; *n* is number of patients.

^a^ Mean (standard deviation, SD).

^b^ Median (interquartile range, IQR).

^c^ Is number and proportion of patients *n* (%). *P*-value for comparison completed vs. discontinued early by *t*-test or Chi square.

**Figure 1:**
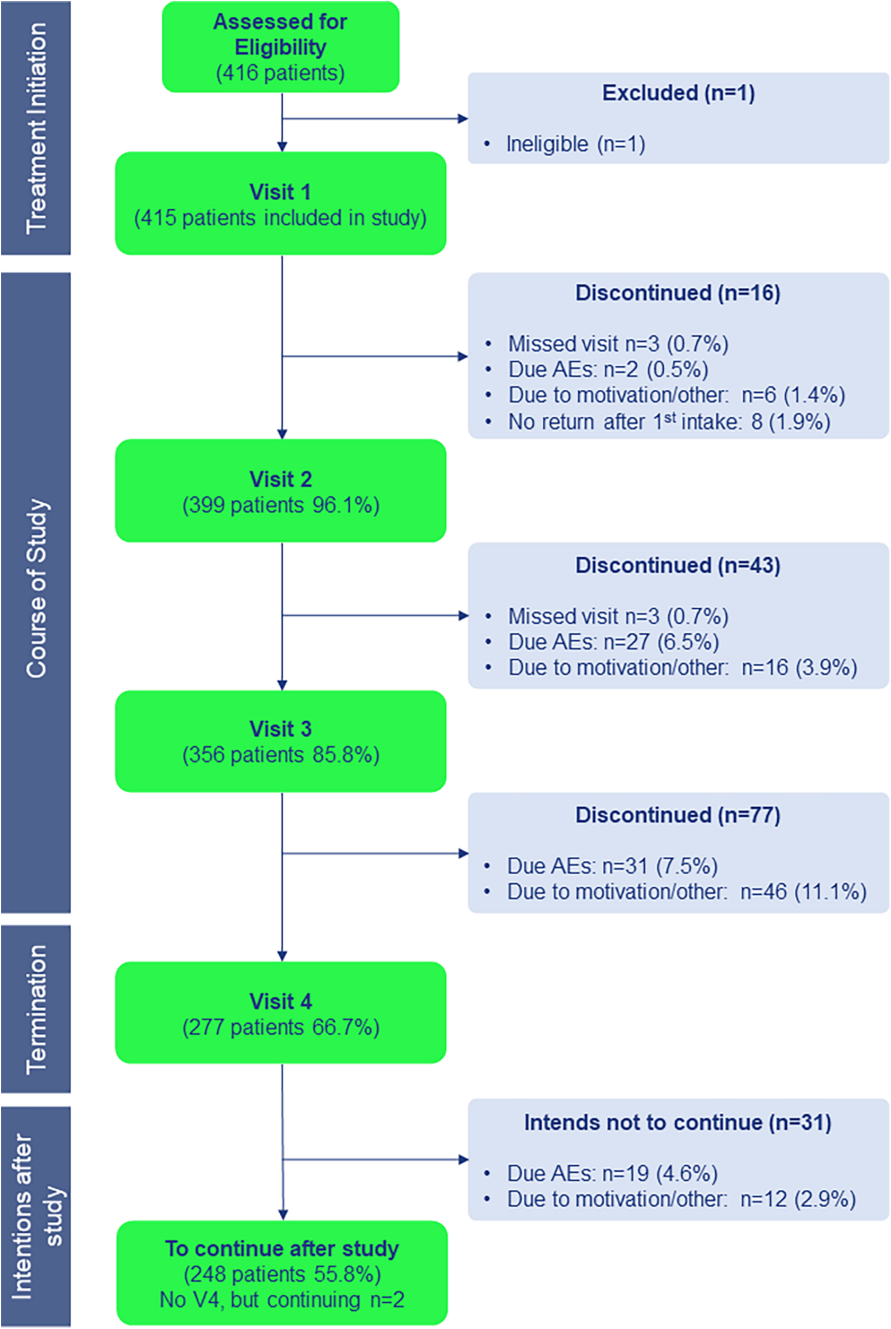
Study flow and follow-up. V1 to V4: visits 1 is day 1, V2 is phone call after 1 week, V3 is visit after 3 months, V4 is visit after 1 year of start treatment with HDM SLIT-tablet; after study completion patients were asked if they intended to continue treatment after the study; *N* is number of patients; AEs is adverse events. Percentages represent proportion of patients that were included (*N* = 415) in the study.

The majority of patients completed the study (Figure 1). Younger patients and patients treated by general practitioners (GP) were more likely to discontinue treatment. Other categories did not differ significantly.

### 4.2 Safety

**Figure 2:**
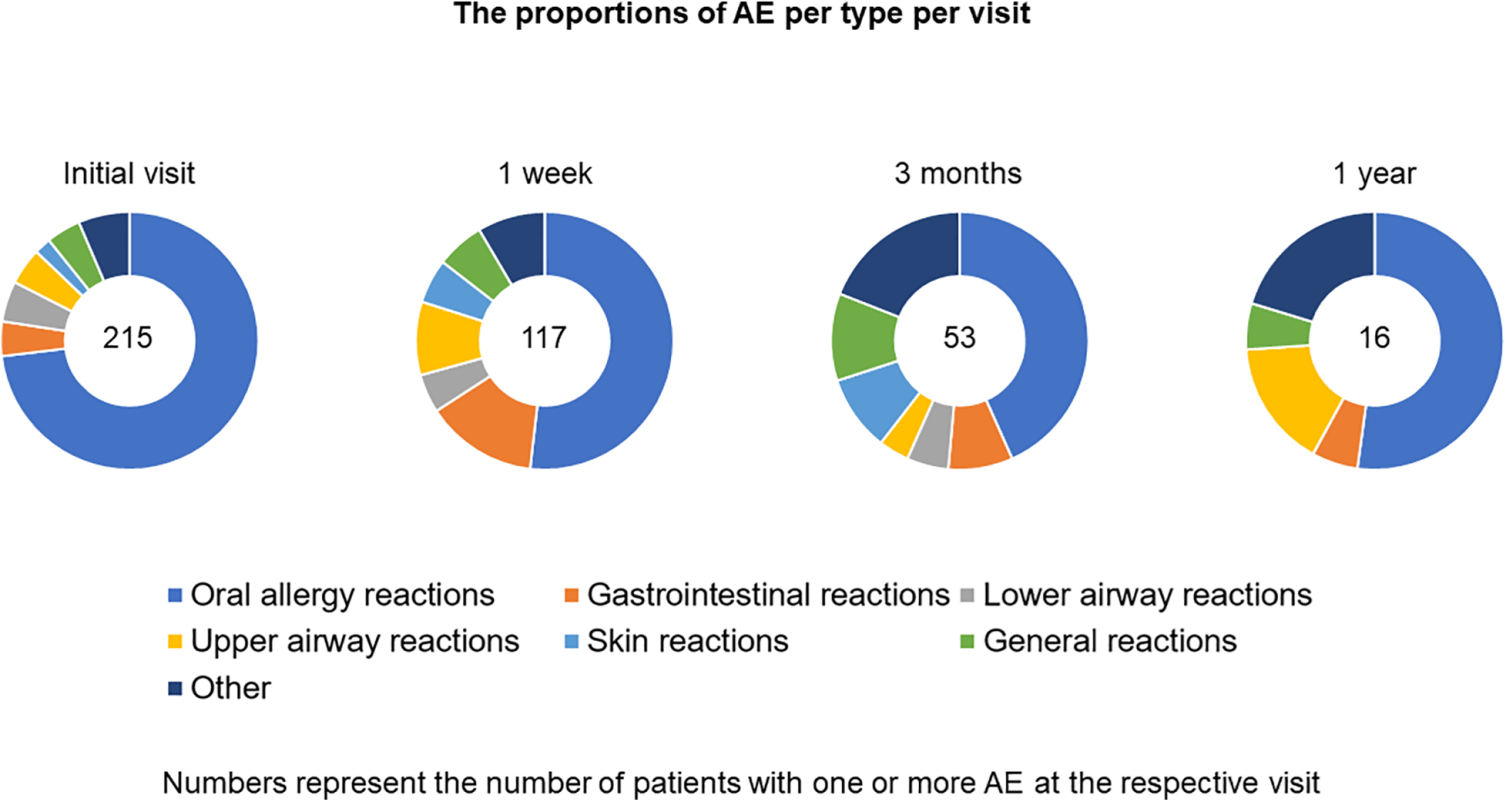
Adverse events per type of adverse event.

**Figure 3:**
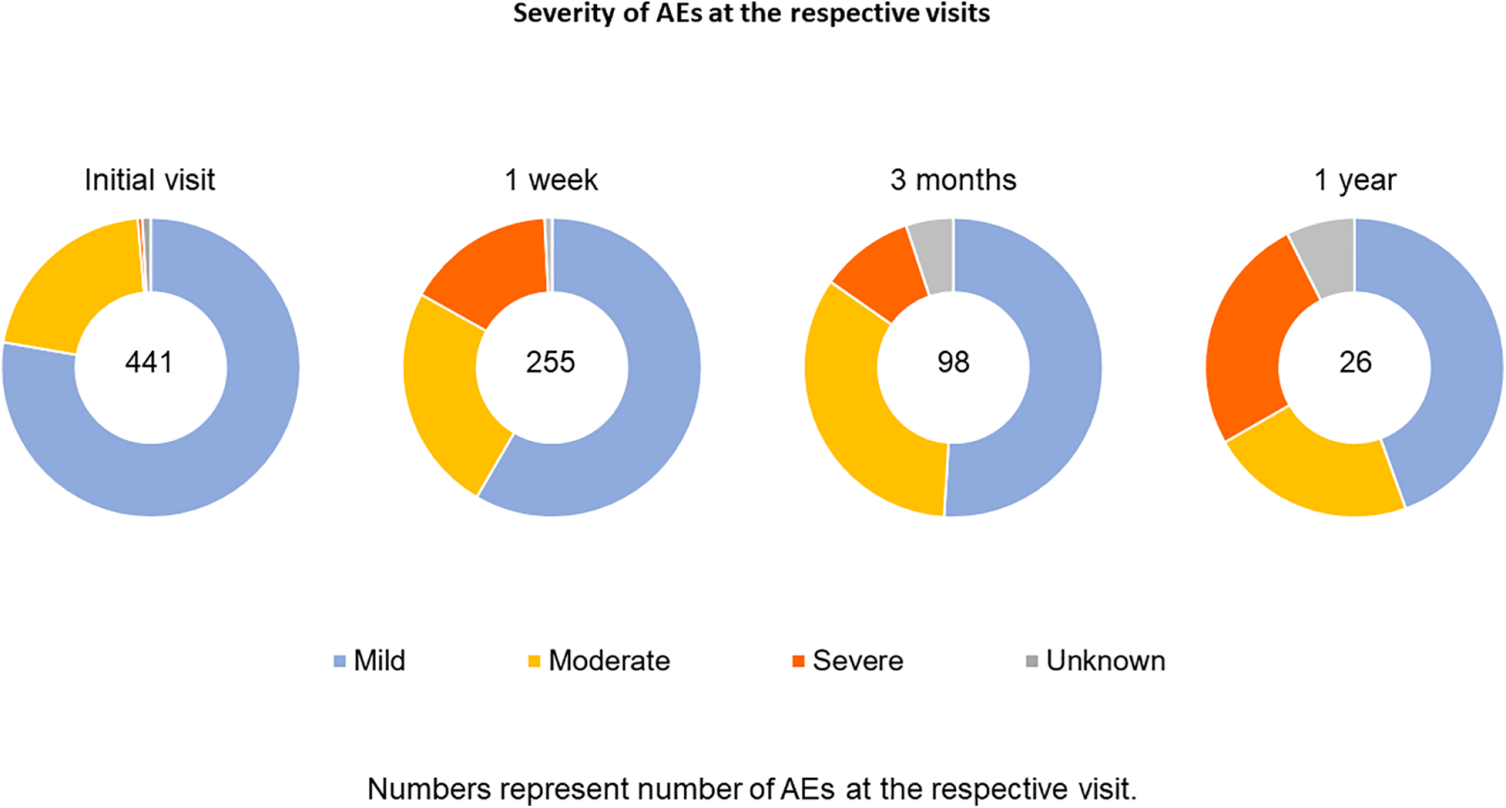
Severity of possibly related adverse events per visit.

AEs were frequent, but mostly local and mild (Tables 2, 3 and Figures 2, 3). In total 970 AEs were reported. Eight hundred and thirty-six (836; 86.2%) of AEs in 270 (65.1%) patients were possibly related to the study drug. A hundred and two (10.5%) AEs were unlikely to be related and 32 (3.3%) AEs had no relatedness assigned by the treating physician. Further analyses focused on related AEs.

**Table 2:** Treatment related AEs.

	Over total study period	Initial visit	1 week	3 months	1 year
Patients attending visit	*N* = 415	*n* = 415	*n* = 397	*n* = 356	*n* = 277
Subjects with any AE^a^	270 (65.1)	215 (51.8)	117 (29.5)	53 (15.0)	16 (5.8)
Subjects with any moderate or severe AE^a^	96 (23.1)	42 (10.1)	37 (9.3)	22 (6.2)	6 (2.2)
AEs per patient^b^	3 (2;5)	2 (1;3)	2 (1;3)	2 (1;2)	1 (1;1,25)
Subjects per specific category of AEs^a^					
Oral allergy reactions^a^	243 (58.6)	203 (48.9)	85 (21.4)	32 (9.1)	10 (3.6)
Gastrointestinal reactions^a^	39 (9.4)	12 (2.9)	23 (5.8)	6 (1.7)	1 (0.4)
Airway reactions^a^	51 (12.3)	27 (6.5)	20 (5.0)	7 (2.0)	3 (1.1)
- Lower airway reactions^a^	26 (6.3)	14 (3.4)	8 (2.0)	4 (1.1)	0 (0.0)
- Upper airway reactions^a^	35 (8.4)	13 (3.1)	15 (3.8)	3 (0.8)	3 (1.1)
Skin reactions^a^	20 (4.8)	6 (1.4)	9 (2.3)	7 (2.0)	0 (0.0)
General reactions^a^	28 (6.7)	12 (2.9)	10 (2.5)	8 (2.3)	1 (0.4)
Other^a^	59 (14.2)	18 (4.3)	14 (3.5)	14 (4.0)	4 (1.4)

^a^ *n* (%) is number and proportion of patients.

^b^ median (interquartile range); AE, adverse event.

**Table 3:** Severity, outcome & study drug adjustment for treatment related AEs per visit.

AE characterization by	Initial visit	1 week	3 months	1 year
Total number of AEs per visit moment	441	255	98	26
Severity of AEs				
Mild	343 (77.8)	149 (58.4)	50 (51.0)	12 (46.2)
Moderate	92 (20.9)	63 (24.7)	33 (33.7)	6 (23.1)
Severe	2 (0.5)	41 (16.1)	10 (10.2)	7 (26.9)
Unknown	4 (0.9)	2 (0.8)	5 (5.1)	1 (7.7)
Course/Outcome				
Recovered	390 (88.4)	179 (70.2)	75 (76.5)	19 (73.1)
Recovered with residual symptoms	3 (0.7)	5 (2.0)	2 (2.0)	0 (0)
No recovery	8 (1.8)	23 (9.0)	4 (4.1)	1 (3.8)
Fatal	0 (0)	0 (0)	0 (0)	0 (0)
Unknown	40 (9.1)	48 (18.8)	17 (17.3)	6 (23.1)
Study drug adjustment				
No	428 (97.1)	193 (75.7)	76 (77.6)	16 (61.5)
Temporary interrupted	6 (1.4)	15 (5.9)	8 (8.2)	0 (0)
Stopped	6 (1.4)	47 (18.4)	14 (14.3)	9 (34.6)
Unknown	1 (0.2)	0 (0)	0 (0)	1 (3.8)

AEs, adverse events. Number of AEs and proportion (%) of AEs ongoing at respective study visits. First administration at initial visit. For reasons of comparability only related AEs with complete assessment of severity, course/outcome, and study drug assessment are represented (820 AEs) in this table.

Of AEs reported 563 (67.3%) were mild, 196 (23.4%) moderate and 64 (7.7%) severe. Most frequently reported were oral allergy reactions oral paresthesia (11.0%), throat irritation (10.2%) and oral pruritus (8.1%) (Table 4).

**Table 4:** Ten most frequent adverse events by preferred term and classification.

Preferred Term	Frequency of AE (number & percentages of events)
Paresthesia oral	92 (11.0%)
Throat irritation	85 (10.2%)
Oral pruritus	68 (8.1%)
Ear pruritus	60 (7.2%)
Pharyngeal swelling	30 (3.6%)
Pharyngeal paresthesia	25 (3.0%)
Mouth swelling	25 (3.0%)
Lip swelling	18 (2.2%)
Swollen tongue	18 (2.2%)
Dyspnea	16 (1.9%)

Related adverse events defined as any possibly related adverse event. Percentages are percentages of related adverse events (836 AEs).

Eighty-one (9.7%) AEs resulted in treatment discontinuation, 29 (3.5%) in temporary discontinuation and the majority of AEs 724 (86.6%) had no effect on treatment. The most prevalent severe AEs leading to discontinuation were swollen tongue (0.7%), mouth swelling (0.6%) and nausea (0.5%). Most patients fully recovered; most AEs were reported before the final visit and no longer reported at the end of the study (81.1%). A small number of AEs (10, being 1.2% of possibly related AE's) belonged to the patients with “some symptoms” during the study visits and 36 AEs (4.3%) were reported from the patients who did not report recovery during the 4 study visits. For 112 (13.4%) events this was unknown (Table 3).

Twenty-four (24) SAEs were reported. Only one SAE (angioedema) was related to the study drug. It occurred at the 3-month visit. The patient recovered fully after discontinuation of treatment.

The percentage of patients reporting AEs decreased from 51.8% at day 1–5.8% after 1 year in those remaining on treatment. The odds that a patient had any AE compared to having any AE(s) directly after first intake, decreased after 1 week [Odds Ratio (OR) = 0.33, *P *< 0.0001], 3 months (OR = 0.13, *P *< 0.0001), and 1 year (OR = 0.04, *P *< 0.0001). These results did not change after correction for potential confounders.

A higher frequency of AEs was observed in patients that were non-compliant with treatment at the initial visit (*P *< 0.0001) and final visit (*P *= 0.0004) (Table 5). Furthermore, a tendency towards a higher frequency of AEs was observed at the 3 months visit (*P *= 0.053). As compliance was estimated by the treating physician it was not assess during the call at 1 week. All in all, treatment compliance and the frequency of AEs were associated. No differences in frequencies of AEs were observed between patients that had multiple (major) allergies, or concomitant asthma or that are being treated with other immunotherapies concurrently, compared to patients without the respective capacities (all *P *> 0.05).

**Table 5:** Specific patient groups and frequency of adverse events per visit.

Patient group		Initial visit		*P*-value	1 week		*P*-value	3 months		*P*-value	1 year		*P*-value
		With	Without		With	Without		With	Without		With	Without	
Multiple allergies	N	328	87		311	85		276	77		217	60	
	AEs	1 (0;2)	1 (0;2)	0.922	0 (0;1)	0 (0;0)	0.100	0 (0;0)	0 (0;0)	0.224	0 (0;0)	0 (0;0)	0.775
Multiple major allergies	N	259	156		245	151		220	133		171	106	
	AEs	1 (0;2)	1 (0;2)	0.536	0 (0;1)	0 (0;1)	0.671	0 (0;0)	0 (0;0)	0.216	0 (0;0)	0 (0;0)	0.325
Concomitant asthma	n	150	265		143	253		132	221		105	172	
	AEs	0 (0;2)	1 (0;2)	0.145	0 (0;1)	0 (0;1)	0.942	0 (0;0)	0 (0;0)	0.312	0 (0;0)	0 (0;0)	0.620
Compliance (>80%, 50–80%, <50%)	n	384	4/7		n/a			323	18/10		240	18/14	
	AEs	0 (0;1)	0.5 (0.0;2.2)	*P* < 0.0001				0 (0;0)	0 (0;0)	0.053	0 (0;0)	0 (0;0)	0.0004
			2 (1.5;3)						0 (0;1)			0 (0;0.75)	
Concurrent immunotherapy	n	13	402		12	384		10	343		6	271	
	AEs	0 (0;1)	1 (0;2)	0.452	0 (0;1.25)	0 (0;1)	0.297	0 (0;0)	0 (0;0)	0.607	0 (0;0)	0 (0;0)	0.262

“AE” is adverse events and “*n*” the number of patients. AE are reported as median (25% percentile; 75% percentile). *P*-values by tested for difference with Mann–Whitney tests. Patients having the respective capacity listed in the first column are in the columns “with” and those that do not have this capacity are listed in columns “without” per visit. Multiple allergies are defined as having 1 or more allergies next to house dust mite allergy. Major allergies are defined as allergies that are moderate or severe in nature. Good compliance was defined as taking medication every day during 80% or more of the days.

### 4.3 Treatment adherence: persistence and compliance

In total 138 patients discontinued the treatment (60 because of AEs, 78 due to motivation/other reasons, i.e., lost to follow up 38, did not return after first intake 8, lack of motivation 4, non-compliant 4, moved houses 3, anxiety for treatment & psychological issues 4, forgetting medication 3, co-payment 2, want to become pregnant 2, bankruptcy hospital 2, missed final visit 2 (but indicated to continue after study), other 6). Treatment persistence was, therefore, 66.7% overall (Figure 1). Subsequently, 248 (59.8%) patients intended to continue treatment for another 2 years in line with the guidelines. A high treatment compliance, i.e., at least 80% of medication taken daily, was observed for those that persisted with treatment. High compliance was reported for 96.7%, 91.5% and 86.6% of patients at week 1, 3 months and 1 year, respectively.

### 4.4 Perceived treatment tolerability and treatment satisfaction

Of patients that answered, the majority (72.8%) was satisfied/very satisfied with the treatment, and 27.2% unsatisfied/very unsatisfied. The majority of physicians agreed (80.0% and 20.0%, respectively) (Table 6). Treatment tolerability was reported to be well to very well according to 74.5% of patients and 80.1% according to their physicians (Table 6).

**Table 6:** Final evaluation of treatment satisfaction and tolerability.

Treatment tolerability	Very well	Well	Moderately	Poorly tolerated	No response
Patient (*n* = 357)	121 (33.9)	145 (40.6)	28 (7.8)	63 (17.6)	58
Physician (*n* = 362)	117 (32.3)	173 (47.8)	44 (12.2)	28 (7.8)	53
Treatment satisfaction	Very satisfied	Satisfied	Not satisfied	Very dissatisfied	No response
Patients (*n* = 356)	85 (23.9)	174 (48.9)	78 (21.9)	19 (5.3)	59
Physicians (*n* = 360)	84 (23.3)	204 (56.7)	63 (17.5)	9 (2.5)	55

*n* is number of patients. Frequencies of a selected answer and proportions (%) of total responding patients and observing physicians, respectively.

### 4.5 Effectiveness of the treatment

#### 4.5.1 CARAT

**Figure 4:**
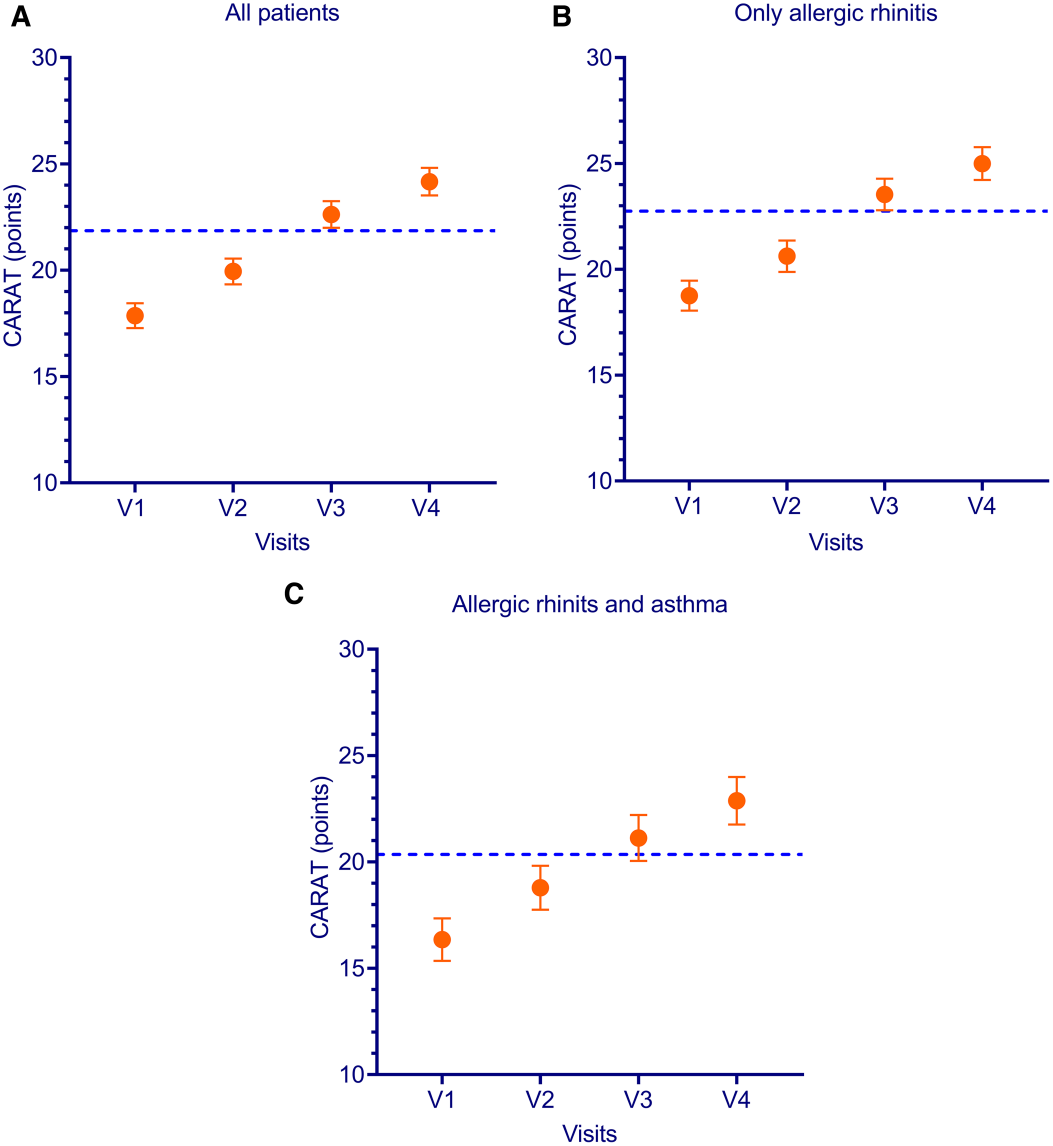
CARAT scores per visit and per allergic rhinitis and asthma status. CARAT is combined allergic rhinitis and asthma test; V is visit; MCID is minimal clinical important difference. Dotted line indicates MCID of +4 points compared to baseline. All patients (**A**) patients with rhinitis but without asthma (**B**) patients with Allergic rhinitis and asthma (**C**).

When CARAT scores at 1 week, 3 months and 1 year of treatment were compared to pre-treatment values using linear multilevel modelling, scores increased with 2.14 (95% CI 1.59–2.69, *P *< 0.0001), 4.69 (4.11–5.26, *P* < 0.0001), and 5.92 (5.30–6.55, *P *< 0.0001), respectively. The accepted MCID is 4 points. Therefore, these results indicate that most patients improved after 3 months and 1 year of treatment (Figure 4, Supplementary Table E1).

When separately analyzing the AR only and AR + AA subgroups, the AR + AA group had a numerically larger improvement (6.90 vs. 5.38 points; *P *= 0.06) after 1 year of treatment (see Supplementary Table E1).

#### 4.5.2 Symptomatic medication

The use of any symptomatic medication decreased significantly from 96.1% of the patients taking one or more symptomatic medications (antihistamine, nasal corticosteroids, etc.) at day 1 of HDM SLIT-tablet treatment to 85.9% at 1 week, 83.0% at 3 months and 75.5% at 1 year of treatment, respectively.

## 5 Discussion

In this observational study, safety, tolerability, satisfaction, compliance, and clinical effectiveness were assessed for HDM SLIT treatment in Dutch daily practice. AEs observed are mostly mild to moderate and decrease in frequency with treatment duration, confirming HDM SLIT-tablet safety. Moreover, symptomatic medication uses decrease, and CARAT scores improve with treatment, indicating clinical effectiveness thus confirming safety and clinical efficacy previously established in trials (14–16). In addition, compliance is high in treatment-persistent patients and both patients and physicians assess treatment to be tolerable and satisfactory.

In this study, 271 (65.3%) patients experienced AEs. Oral allergy reactions (58.6%) were most frequently observed, followed by airway complaints (12.4%), and GI reactions (9.4%). In the phase III trial by Demoly et al. (14) 67% of patients on 12 SQ-HDM experienced AEs comprising: oral pruritis (20%), throat irritation (14%) and mouth oedema (8%); indicating similarity in frequency and location of AEs in clinical trial and in daily-practice setting. The proportion of AEs experienced by patients in our real-life study is higher than in France (32%) but lower than in Scandinavia (80%) (28, 29). Possible explanations for the observed differences may include differences in study design and environmental factors such as climate and time spent outdoors (30, 31).

Furthermore, specific patient groups that potentially have an elevated risk for having AEs were compared to patients that did not have the respective potential elevated risk. There were no differences found between patients having multiple (major) allergies or concomitant asthma compared to patients with a mono-allergy or without asthma, respectively. The number of patients on concomitant immunotherapy was low. Concomitant immunotherapy was also not associated with increased numbers of AEs. However, more AEs were found in patients with sub-optimal and poor compliance compared to compliant patients (Table 5).

The precise mechanism of action of AIT may still be unknown but it has been broadly researched. It has been hypothesized that low doses of allergens stimulate the pro-allergenic response, while at higher doses suppression of the allergenic response occurs. Therefore, high doses of allergens are administered in AIT. It is thought that tolerogenic dendrite cells and other cells are induced in the oral mucosa of the patient, which, subsequently, leads to reduction of allergen-specific T-helper-2 cells and induction of Th1 cells and regulatory T and B cells. The Treg cells subsequently stimulate B cells to produce allergen specific IgG and IgA that compete with IgE and thus inhibit the IgE mediated release of histamines from mast cells and basophils (11, 32).

CARAT questionnaires were used to monitor treatment as recommended by Dutch guidelines (33). The CARAT questionnaire had been validated by Fonseca et al. by comparing it to ACQ, GINA, ARIA, VAS scores (upper and lower airways) and physician assessments (25). In their validation they found a test-retest ICC of 0.82, which is higher than previously reported for ACT and also the Guyatt's responsiveness index was slightly higher than ACQ7 (25). The CARAT questionnaire could thus be compared to pre-existing questionnaires with respect to asthma. With respect to AR it could be compared to VAS scores of the upper airways and physician assessments (25). CARAT was,subsequently, validated in the Netherlands (24, 25). All in all, CARAT has been extensively validated and is the only questionnaire to assess both asthma and AR (24, 25, 27). Furthermore, the MCID, the minimal amount of change to be clinically meaningful for a patient, was determined to be a change of at least 4 points in CARAT scores (24, 34). Therefore, *post hoc* analyses were conducted to assess both statistically significant changes in CARAT scores compared to baseline and to determine whether these changes were likely to be clinically meaningful, i.e., effect size equal or larger than the MCID. CARAT scores increased significantly and clinically meaningful after 3 months (> 4 points) and 1 year (6 point) treatment compared to baseline. This shows clinical effectiveness in real life and confirms the previously established clinical efficacy (14–16).

In real life, patients often do not fill out their prescriptions as advised, while AIT treatment needs to be persisted for 3 years (23, 35). In the past, major issues were reported with SLIT drops compliance in the Netherlands (36). Only 7% of patients were found to consistently and timely pick up refills (36). In the present study, compliance (defined as 80%-100% of SLIT-tablets taken daily) was excellent: 96.7% at 1 week, 91.5% at 3 months and 86.6% at 1 year, respectively. This is in line with recent studies. In a Swedish-Danish study SLIT-tablet compliance was 93.2% at 1-year (37). Moreover, data from the Danish prescription register showed a compliance rate of 53% and 57% for SLIT-tablet and SCIT, respectively, after 3-years treatment (29). Furthermore, compliance in a recent Dutch grass pollen SLIT-tablet study was 76% (43) Possible explanations for the observed differences include: increased attention on compliance, the definition of compliance, different study populations, and differences in SLIT type (drops vs. tablets) (35). Daily intake of tablets may be cumbersome in the beginning but after a period of time, in particular doing it in a routine way, e.g., before diner, it becomes a habit and less of a burden (26, 38).

Ways to improve compliance include more patient visits and selecting patients dedicated to persisting treatment (35, 39). For example compliance in clinical trials is generally higher than in real-life studies (40). In addition, solid patient education on SLIT-tablet treatment may help (35, 39). Part of patient education may be reassuring patients, that AEs will decrease over time as validated by this study (35, 39). Moreover, if AEs occur, adding symptomatic medication may be considered (23).

Discontinuation was higher in this real-life study compared to the phase III trial by Demoly et al. (14). 14.5% of patients stopped treatment due to AEs and 18.3% patients stopped because of motivational/other reasons vs. 4.1% and 6.6% in the trial, respectively. However, discontinuation was in line with a recent Dutch real-life grass pollen SLIT-tablet study, which reported discontinuation in 9.8% of patients due to AEs and 15.3% because of other reasons (41). Possible explanations may be the more intensive follow up of patients in trials and variations in patient populations (35).

Patients and their physicians were asked about satisfaction and tolerability of treatment when they discontinued or completed the study. Responding patients (74.5%) and physicians (80.1%) reported treatment to be well to very-well tolerable. Moreover, most patients (72.8% of responders; 62.4% of all patients) and their physicians (80.0% responders; 69.4% for all patients) were satisfied with treatment. This is in line with previously reported satisfaction for SLIT-tablet therapy (41, 42).

### 5.1 Strengths and limitations

The study was limited to 1 year. Therefore, AEs that developed subsequently may be missed. Since previous studies have shown that most (serious) AEs occur at the beginning of treatment (43, 44) and AEs decrease over time from 51.8% at day 1 to 5.8% after 1 year in this study, we consider this risk to be low.

Out of 415 patients, 138 did not complete the study. Discontinuation of treatment is a common problem when administering SLIT (35, 39). This might have had impact on our outcomes i.e., there could be an underestimation of the safety and tolerability effects and an overestimation of effectiveness. Nonetheless, no major differences were observed in baseline characteristics of patients that continued vs. those that discontinued treatment, indicating that outcomes are representative for all patients.

Finally, treatment compliance as estimated by physicians is high (≥80% daily intake by ≥85% of patients at every visit during the study). It would be of interest to know if poor compliance precedes discontinuation or that patients discontinue without first becoming non-compliant. Indeed, not being compliant was associated with having more AEs (Table 5). However, the low number of non-compliant patients at each visit makes it impossible to test and confirm this hypothesis.

### 5.2 Implications for clinical practice

HDM SLIT-tablet should only be prescribed to motivated patients. It is also important to emphasize the high likelihood of developing one or more AEs (>65%). At the same time, it is essential to address that most AEs are mild and likely resolve with time. However, there is still a chance that patients will stop treatment because of AEs.

### 5.3 Conclusions

Our study confirms that 12 SQ-HDM SLIT-tablet (ACARIZAX®) is a safe and well-tolerated treatment for HDM AR in daily clinical practice.

Adverse events are common but are mostly mild and decrease during the first year. Clinical scores (CARAT) improve, and symptomatic medication use decreases with treatment duration. If patients continue the therapy, compliance rates are high and treatment satisfaction is good. However, with a stopping rate of 14.5% and 18.3% due to adverse events and motivational reasons, compliance remains the main concern when starting HDM SLIT-tablet treatment.

## Data Availability

The datasets presented in this study can be found in online repositories: https://catalogues.ema.europa.eu/node/3903/administrative-details. The names of the repository/repositories and accession number(s) can be found in the article/Supplementary Material.
