# Supplementary material for: House dust mite sublingual allergen immunotherapy tablet is safe and well-tolerated in Dutch clinical practice
Source: Front Allergy. 2024 Feb 29;5:1355324. doi: 10.3389/falgy.2024.1355324 (PMC10937523; doi:10.3389/falgy.2024.1355324)
Supplement: Supplementary file 1 [file Table1.docx]

Supplementary Material

**Definitions of adverse event assessment**

Relatedness was defined as either ‘’Possible’’: a causal relationship is conceivable and at least reasonably possible; or ‘’Unlikely’’: the event is most likely related to a different etiology than the medicinal product. AEs with unclear causality were categorized as possibly related. Severity was defined as ‘’Mild’’: No or transient symptoms, no interference with the patient’s daily activities; ‘’Moderate’’: Marked symptoms, moderate interference with the patient’s daily activities; or ‘’Severe’’: Considerable interference with the patient’s daily activities, unacceptable.

**Details on AE statical descriptions**

Safety data were described by the number and proportion of patients with any AE, overall and per type of AE (oral allergy reactions, GI reactions, airway reactions, lower airways reactions, upper airway reactions, skin reactions, general reactions, or other reactions) and the number of patients with any moderate or severe AE. The median number (with IQR: 25^th^ and 75^th^ percentile Q1:Q3) of AEs experienced per patient in those experiencing any AE was also calculated. All analyses were performed separately per study visit.

**TABLE E1** CARAT scores improved in all AR patients with or without asthma during HDM SLIT-tablet treatment

| **Visit** | **initial** | **1 week call** | **3 months** | **1 year** |
| --- | --- | --- | --- | --- |
| *All patients #* | 415 | 399 | 357 | 277 |
| CARAT reported # | 413 | 379 | 342 | 266 |
|  |  |  |  |  |
| CARAT mean (SD) | 17.84 (6.08) | 19.94 (6.01) | 22.62 (5.90) | 24.17 (5.39) |
|  |  |  |  |  |
| *Patients no asthma* |  |  |  |  |
| CARAT reported # | 265 | 238 | 210 | 160 |
| CARAT mean (SD) | 18.97 (5.77) | 20.59 (5.80) | 23.40 (5.71) | 24.86 (5.22) |
|  |  |  |  |  |
| *Patients with Asthma* |  |  |  |  |
| CARAT reported # | 148 | 141 | 132 | 106 |
| CARAT mean (SD) | 15.87 (6.14) | 18.80 (6.21) | 21.31 (6.00) | 23.03 (5.50) |

CARAT: Combined Allergic Rhinitis and Asthma Test; # Number of patients; mean and standard deviations (SD) per visit for all patients, for patients with asthma and for patients without asthma, respectively. **P* difference versus baseline<0.0001 and CARAT difference>4 points.
